# Formation of Refractory Monomethylmercury Pools by Particle Adsorption

**DOI:** 10.1021/acsestwater.6c00243

**Published:** 2026-09-19

**Authors:** Sonja Gindorf, Abhinav Bharadwaj Tirunaghari, Charlotte Haugk, Alyssa Azaroff, A. Britta K. Sannel, Sofi Jonsson

**Affiliations:** † Department of Environmental Science, Stockholm University, 114 18 Stockholm, Sweden; ‡ Bolin Centre for Climate Research, Stockholm University, 114 18 Stockholm, Sweden; § Department of Physical Geography, Stockholm University, 114 18 Stockholm, Sweden

**Keywords:** desorption, partitioning, mercury, methylmercury, terrestrial transport, sub-Arctic

## Abstract

Adsorption of mercury (Hg) onto particles is known to limit Hg availability for important biogeochemical processes, including the methylation of Hg and biological uptake of monomethylmercury (MMHg). However, the extent to which particle adsorption forms refractory Hg pools, i.e., pools not readily available for desorption, remains largely unexplored. Here, we present novel data on the formation of refractory particle-bound MMHg in slurries prepared from environmental soils and waters representing a gradient from terrestrially influenced to marine systems. Refractory pools were quantified from differences in equilibrium partitioning between adsorption (up to 48 h) and desorption (up to 8 weeks) using an isotopically labeled MMHg tracer. Refractory particle-bound MMHg pools formed in all soil–water slurry combinations. The organic carbon content in soil and water increased and decreased partitioning coefficients, respectively, while effects on refractory particulate MMHg formation were less pronounced. These findings demonstrate the importance of considering refractory MMHg pools when predicting the fate and associated environmental risks of MMHg exported from terrestrial systems.

## Graphical abstract

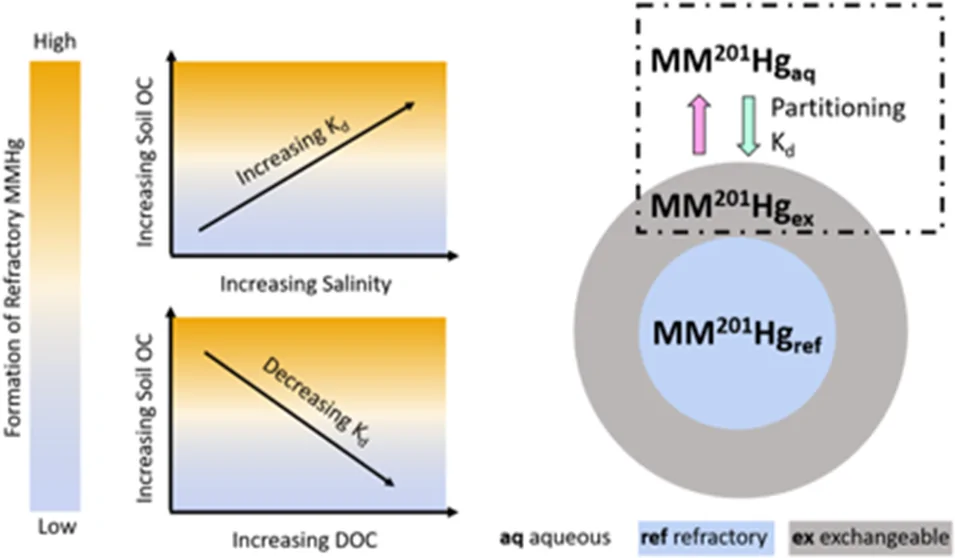

## Introduction

Large amounts of mercury (Hg) are transported from land, via streams and rivers, to lakes and coastal systems, where it can bioaccumulate in aquatic food webs.^1,2^ Particles act as an important vector for this transport,^3^ but to what extent particle-bound Hg is available for desorption and thereby contributes to the dissolved Hg pool available for biological uptake is largely unknown.

Among the different chemical forms of Hg found in the environment, monomethylmercury (CH_3_Hg^+^, hereon referred to as MMHg) is particularly concerning to human and environmental health due to its neurotoxicity and ability to biomagnify.^4^ MMHg entering the food webs of lakes and coastal waters stems from terrestrial sources and/or *in situ* production through microbial methylation of inorganic divalent Hg (Hg^2+^, hereon referred to as Hg^II^). Both Hg^II^ and MMHg have a strong affinity to particles, which can limit their availability for methylation and bioaccumulation, respectively.^5^ In natural waters, concentrations of particle-bound Hg (Hg^II^P, MMHgP) vary temporally and spatially and can represent the main form of MMHg in the water in some systems.^3,6–8^ In addition to lowering the amounts of Hg^II^ and MMHg available in the dissolved phase, adsorption of Hg to particles may result in the formation of refractory Hg^II^ and MMHg pools, e.g., pools of Hg^II^ and MMHg which are not readily available for desorption.^9–12^ Testing the formation of refractory particulate Hg species is, however, challenging, as all natural matrices contain Hg, which makes it impossible to distinguish the source of Hg during partitioning experiments. In this study, we focused on the formation of refractory MMHg pools by particle adsorption.

The existence of refractory Hg^II^P and/or MMHgP pools has been previously indicated in both field and experimental studies. Isotopic Hg fractionation data from the Mackenzie River basin in Canada, one of the largest Arctic rivers, revealed that aqueous HgP had a signature similar to that of terrestrial soil and vegetation, whereas the dissolved Hg pool had an isotopic signature of atmospheric sources.^8^ These results support the idea that the dissolved and particulate Hg pools are derived from different sources and that the two pools are not readily exchanged during transport in the river. In another study, an added MMHg tracer remained mainly methylated over the course of a six-week-long incubation period in various sediment and soil slurries, suggesting that refractory pools were formed and remained unavailable for demethylation.^12^ Furthermore, based on field data from the Atlantic Ocean and a model simulating the partitioning of Hg to marine particles, Lamborg et al. (2016) suggested that remineralization of marine particles was necessary for HgP to enter the dissolved pool.^10^

Accounting for refractory HgP has important implications for understanding the environmental fate of Hg loads entering coastal systems. This has, for example, previously been highlighted in a modeling study by Zhang et al. (2015).^9^ The authors compared two extreme scenarios for riverine HgP where either all Hg was bound refractory (not available for desorption) or in a reversible equilibrium (continuous partitioning between the solid and aqueous phases). They demonstrated that this differentiation has great implications for the fate of riverine Hg. In the reversible equilibrium scenario, 40% of Hg is exported to the open ocean, 47% is evaded from the coastal ocean to the atmosphere, and only 13% is buried in shelf sediments. In contrast, if all riverine particulate Hg was refractory, over 90% of the riverine particulate Hg would be buried in shelf or estuary sediments.^9^ Especially in the Arctic, improved predictions of the fate of riverine MMHgP are needed to implement environmental management measures protecting the health of wildlife and humans as Arctic ecosystems are drastically changing. The Arctic is warming almost four times faster than the global average temperature increase.^13^ This rapid warming drives the thaw of permafrost, which stores large amounts of Hg in the upper soil.^14^ How far and in what form Hg released from thawing permafrost can be transported and whether it may be available to bioaccumulate and biomagnify in Arctic food webs are unknown. Understanding the dynamics driving the formation of refractory HgP and MMHgP pools can help improve predictions of the form in which Hg may be released and exported from thawing permafrost.

Adequate experimental data on refractory pools of particle-bound Hg species are still missing and needed to account for the refractory pools of Hg in biogeochemical Hg models. Currently, HgP is either modeled based on reversible partitioning coefficients^15^ or remineralization of particles is needed for riverine terrestrially derived HgP to enter the dissolved pool.^16^ We also lack information regarding whether soil and water characteristics can be useful proxies to gain insights into how much of the exported Hg is transported in a refractory phase. Here, we use an isotopically labeled MMHg tracer to study the discrepancy between adsorption and desorption partitioning coefficients at equilibrium to quantify the formation of refractory MMHg pools associated with terrestrial particles. We combine solids and waters differing in their characteristics, with respect to, e.g., organic carbon content, salinity, and pH, to provide experiment-derived estimates for the formation of refractory particulate MMHg.

## Methods

### Materials

Three soil types (peat, stream mineral, and mineral) and three water types (pond, stream, and fjord) were collected in northern Sweden and Norway. Differences in the physicochemical properties of these waters (Table S1) and soils (Table S2) affect the properties of the slurries used in our experiments (Table S3). In order to test the role of different environmental endmembers, we chose three solids and waters representative of the gradient from freshwaters to marine systems. All reagents were prepared on a regular basis according to previously described standard protocols.^17^ Three isotopically labeled MM^201^Hg tracer solutions were prepared at concentrations of 50 μg L^–1^ equilibrated with pond, stream, or fjord water for at least 24 h prior to the start of the incubations. An isotopically labeled MM^204^Hg tracer (prepared in 0.5% (v:v) HCl ultrapure water) was used as an internal standard. The chemical speciation of the MM^201^Hg was modeled from the concentrations of MMHg, Cl^–^ (based on salinity), and thiols (calculated as 0.15% * DOC)^18,19^ in prepared tracer solutions and thermodynamic stability constants (Log *K*) of 5.4 for MMHgCl (aq)^20^ and 16.1 to 17.4 for MMHg-DOC (aq).^21^

### Adsorption and Desorption Experiments

A schematic of our experimental setup is presented in Figure S1. To study the partitioning of the MM^201^Hg tracer between the solid and aqueous phases over time, individual slurry incubations were prepared combining the different soils and waters in 50 mL Falcon tubes. For each of the nine water–soil combinations, 17 incubations were prepared: seven time points during adsorption (Table S4), six during desorption (Table S5), and triplicates at the final time point of both phases. All incubations were carried out at controlled temperature (10 °C) in a climate chamber and placed on a rotation table at 6 rotations per minute (rpm).

To account for differences in OC among the soil matrices, slurries were prepared using 0.5 g (wet weight) of peat and mineral soil and 1 g of stream mineral soil, corresponding to dry weights of 0.15 g for the OC-rich peat and 0.45–0.48 g for the mineral and stream mineral samples. Pond, stream, and fjord waters were then added to the Falcon tubes containing the soils to a final volume of 50 mL, as measured using the Falcon tube scale. All slurries were then pre-equilibrated for 24 h. After pre-equilibration, 200 or 400 μL of the respective tracer solution was added, resulting in 10 ng of MM^201^Hg being added to mineral and stream mineral and 20 ng of MM^201^Hg being added to peat incubations. The time recorded for incubation start was the time of tracer addition. The incubations for 0.25 and 0.5 h were manually shaken (as opposed to transferred to the shaking table in the climate chamber) and otherwise processed as described below.

For determining the desorption kinetics of the previously adsorbed MM^201^Hg, the water was removed from all desorption incubations after 48 h (following the same procedure as described below for water removal at the end of an incubation) and new pond, stream, or fjord water was added to the respective samples. The average concentration in the soil at the last time point of the adsorption phase served as a reference for the solid concentration of MM^201^Hg for T0 of the desorption study. The concentration of aqueous MM^201^Hg at the start of the desorption incubation phase (T0) was assumed to be 0 since the process of separating the liquid and aqueous phases to end an incubation takes almost 30 min and desorption is known to occur rapidly.^22^ To account for aqueous MM^201^Hg left in water remaining after removal, the calculated amounts of aqueous MM^201^Hg left after the water removal (following the adsorption phase) were subtracted from the observed mass of aqueous MM^201^Hg at each time point during the desorption phase. For all slurries, the calculated concentration due to dilution of remaining MM^201^Hg after the adsorption time was low in comparison to the observed concentrations during the desorption incubation (0.5–30% of the concentration at the last time point).

To stop the incubations, the sample was first centrifuged at 2000 rpm for 12 min. Then, a silicon tube connected to a 60 mL syringe was used to slowly and carefully remove as much liquid as possible from the Falcon tube without disturbing the solid. The end time of each incubation was defined as the time when solid and liquid phases were separated. After the separation step, the water was filtered through a 0.2 μm polyethylene syringe filter directly into the preservation flasks. Subsamples for MMHg analysis were stored in precombusted (450 °C, >4 h) 40 mL amber glass vials, acidified to 0.5% v:v with trace metal grade (TMG) hydrochloric acid (HCl), sealed with septum caps, and stored at 4 °C until analysis. Samples for dissolved organic carbon (DOC) analysis were transferred into 15 mL Falcon tubes, acidified with HCL to 0.5% (v:v), and stored frozen until analysis. The solid samples remaining in the incubation tubes and were frozen (−20 °C) and subsequently freeze-dried. After noting the dry weight, the samples were stored in the dark at room temperature until analysis.

The stability of the tracer in the different matrices used was tested by incubating each water and soil type separately for up to 8 weeks with five time points and triplicate measurements at the last time point (Figures S3–S4). For the waters, the tracer pre-equilibrated with each respective water was added, and for the soils, the tracer equilibrated with stream water was used.

### Chemical Analysis

Water samples for MMHg analysis were prepared in 40 mL amber glass vials with septum screw caps. Depending on the expected concentration, between 40 and 1200 μL of the sample was added into ultrapure water to reach a concentration between 0.1 and 1 ng L^–1^. The volume of ultrapure water was adjusted to reach a final volume of 30 mL. To all samples, 30 μL of the internal standard MM^204^Hg (concentration ranging from 600 to 800 ng L^–1^) was added. The sample and standard were then left to equilibrate for 1 h. The pH was adjusted to 4.7 using 8N KOH (volume dependent on pH, measured by pipetting a small volume (<1 μL) onto a pH paper with a working range from 4 to 7) and 4 M acetate buffer (225 μL). Finally, 30 μL of sodium tetraethylborate (NaBEt_4_) was added to facilitate ethylation.^23^ Hence, caps were screwed on tightly directly after addition. Each day, calibration standards with natural abundance and the internal standard were analyzed to ensure the performance of the measurement (average accuracy: 99.8%, range: 86–112%). Recovery tests with an addition of 0.5 ng L^–1^ ambient MMHg standard were performed with all water–soil combinations (recovery range: 101–105%, average recovery: 104%, *n* = 9).

MMHg from the soils was extracted via solid–liquid extraction. Depending on the expected concentration based on the time point and water–soil combination, between 50 and 200 μL of a MM^204^Hg tracer (concentration: ca. 1300 ng L^–1^) was added as an internal standard to the whole freeze-dried sample and equilibrated for 1 h. For the extraction, 10 mL of KBr (1.4M), 2 mL of CuSO_4_ (2M), and 10 mL of dichloromethane (DCM, CH_2_Cl_2_) were added to each sample. After 45 min of equilibration, samples were placed on a sample rotor for 45 min (65 rpm) followed by centrifugation for 5 min (3000 rpm). The supernatant was manually recovered into a new 50 mL Falcon tube using a glass Pasteur pipet. After adding 10 mL of ultrapure water, the DCM was purged from the samples with N_2_ gas through a glass Pasteur pipet in a warm water bath (40 °C).^24,25^ The samples were closed tightly and stored at −20 °C until analysis. For analysis, samples were thawed and a subsample was pipetted into ultrapure water in 40 mL amber glass vials to reach a final volume of 30 mL. The samples were further processed in the same way as the aqueous MMHg samples; after adding 225 μL of 4 M acetate buffer and 30 μL of NaBEt_4_, further analytical steps were identical for the two matrices.

The MMHg samples were measured using a Tekran 2700 methylmercury analyzer based on EPA method 1630 coupled to an inductively coupled plasma-mass spectrometer (ICP-MS, Thermo Scientific iCAP Qnova series) through Teflon tubing. The concentrations of MMHg were quantified using isotopic deconvolution.^26^ As naturally occurring stable isotopes of MMHg were used in our experiment, the enriched isotope was measured against a background of ambient MMHg with natural isotopic abundance. Of relevance here is that ambient Hg contains 29.8% ^202^Hg (isotope measured for ambient MMHg), 13.2% ^201^Hg (isotope measured for the added tracer), and 6.8% ^204^Hg (isotope measured for the internal standard). Because tracer-to-ambient Hg ratios may vary among samples, tracer contributions to the ^201^Hg were quantified and required ≥ 4.8% analyte signal to exceed the detection limit.^11^ All samples met this criterion, with 98.3 ± 3.5% (median ± SD) of the ^201^Hg signal originating from ^201^MMHg.

The concentrations of the isotopically labeled tracer solutions were quantified and diluted using reverse isotopic deconvolution (RID) with an ambient MMHg solution (500 ng L^–1^) prepared from a certified 1000 mg L^–1^ MMHg standard (Alfa Aesar). RID measurements of the MM^201^Hg stock solution were carried out at the start of the incubations, and RIDs of MM^204^Hg were prepared at least weekly along with sample analysis.

DOC measurements were performed on a total organic carbon analyzer (TOC-L, Shimadzu, Japan, limit of detection (LOD) of 0.12 mg L^–1^ determined from blanks (3*SD)). The pH was measured using a pH electrode (Metrohm AG, Switzerland).

Soil carbon content (%) was analyzed using an elemental analyzer (Thermo FlashSmart).^27^ We assume the measured total soil carbon to represent the organic carbon (OC) content as <1% was found to be inorganic carbon in similar soils.^27^

Particle surface area was measured using a FlowPrep 060 (Micromeritics). Between 0.1 and 0.8 g of freeze-dried sample was weighed into analysis vials and degassed at 200 °C using a nitrogen gas flow for at least 2 h. The weight was recorded prior to the analysis. Black carbon (21 ± 0.75 m^2^ g^–1^ dry weight) and titanium dioxide (8.23 ± 0.21 m^2^ g^–1^ dry weight) were used as reference materials.

### Quantification of Refractory MMHg

The concentrations of MM^201^Hg measured in the different waters and particles were used to calculate partitioning coefficients (*K*_d_) for the adsorption (*K*_d Ads_, eq 1) and desorption (*K*_d Des_, eq 2) phases. Both adsorption and desorption rates are reported as partitioning between solid and aqueous phases over time. In order to estimate refractory pools, *K*_d Ads_ and *K*_d Des_ differ in one important assumption, which is that during the adsorption phase, all of the adsorbed tracer is part of the *exchangeable pool* (MM^201^Hg that is available for adsorption and desorption). In contrast, during the desorption pool, the adsorbed MM201Hg consists of the *exchangeable* and *refractory pools* (adsorbed MM^201^Hg not available for desorption). Based on this assumption, the concentration of the refractory MM^201^Hg was calculated using eq 3

1 $$K_{d\;Ads}=\left(\frac{{[MM}^{201}{Hg]}_{ex}}{{[MM}^{201}Hg{]}_{aq}}\right)$$

2 $$K_{d\;Des}=\left(\frac{{[MM}^{201}{Hg]}_{ex}+{[MM}^{201}{Hg]}_{ref}}{{[MM}^{201}{Hg]}_{aq}}\right)$$

3 $${[MM}^{201}{Hg]}_{ref}={(K}_{d\;Des}-K_{d\;Ads})\times {[MM}^{201}{Hg]}_{aq}$$

where

*K*_d Ads_ is the adsorption partitioning coefficient.

[MM^201^Hg]_ex_ is the concentration of MM^201^Hg that is available for adsorption and desorption.

[MM^201^Hg]_aq_ is the concentration of MM^201^Hg in the aqueous phase.

*K*_d Des_ is the desorption partitioning coefficient.

[MM^201^Hg]_ref_ is the concentration of adsorbed MM^201^Hg that is not available for desorption.

### Statistics

Averages and standard deviations were computed in MS Excel. All other statistical analyses were computed in JMP 16 Pro. Prior to conducting ANOVA analysis, data were ensured to be normally distributed (Shapiro–Wilk test). When needed, data were log-transformed to meet the normality criterion. The goodness of fit was tested using the Anderson–Darling test. Differences between the waters, soils, slurry combinations, and phases were tested using one-way ANOVA followed by Tukey–Kramer HSD post hoc. The significance level was α = 0.05.

## Results and Discussion

### Adsorption and Desorption Kinetics

The fastest change in *K*_d Ads_ and *K*_d Des_ occurred between the start of the incubation (T0) and the first recorded time point (0.5 and 3 h during adsorption and desorption, respectively) in all water–soil slurry combinations tested (Figure S2). The change in the *K*_d_ over time is presented in Figure 1. From this figure, it can be observed that the *K*_d_ value increases in all slurries within the first day during adsorption. Adsorption equilibrium was reached in most systems within 24 to 48 h, as indicated by the small change in *K*_d Ads_ (Table S4), which is in agreement with previously reported adsorption equilibrium times of MMHg between 1 h and 1 day.^22^ In contrast, desorption is rapid and *K*_d Des_ hardly changes over the course of 8 weeks in slurries with pond and stream waters, while it slightly increases in the slurries with fjord water regardless of the solid type (Figure 1 and Table S5). The stability of *K*_d Ads_ between the last two time points suggests that the adsorption step was long enough to ensure that equilibrium was reached (which is important for our approach when calculating refractory pools). We intentionally chose a shorter incubation time for the adsorption step to avoid excessive demethylation of the added tracer. In a similar fashion, the stability of *K*_d Des_ during the weeks-long desorption phase shows that the *K*_d Des_ values used to calculate the refractory pools were not overestimated due to slow desorption kinetics.

**Figure 1:**
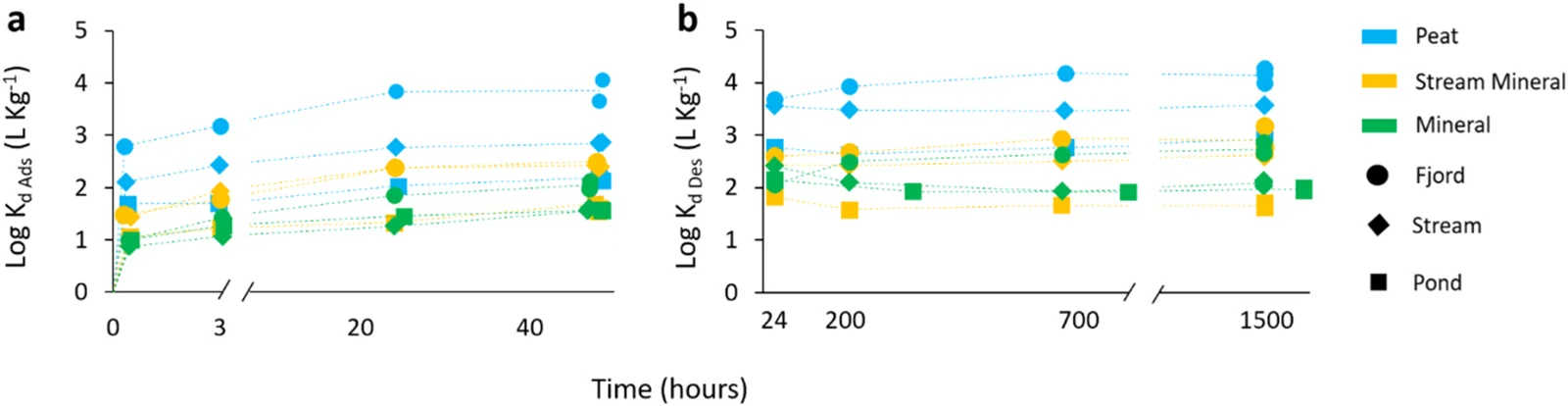
Time series of partitioning coefficients during (a) adsorption (*K*_d Ads_) and (b) desorption (*K*_d Des_). Note the *x*-axis breaks.

### MMHg Stability

During the desorption phase, we observed a decrease of the tracer MMHg concentration in the solid phase in all water–particle slurries (Figure S2D–F) except in the stream–peat–slurry (Figure S2F), where approximately 100% of the tracer was quantified as MMHg in the solid phase over the whole course of the experiment. To test for tracer demethylation, we carried out control experiments in which we monitored the stability of the tracer in each of the waters and soils for up to 8 weeks. We observed that the tracer was stable in the stream water, while over time, it was less stable in the pond water and least stable in the fjord water with >90, ca. 55, and <10% remaining as MMHg at the last time point in the respective waters (Figure S3). In contrast, the tracer was found to be highly stable when adsorbed to the peat and stream mineral soil, while the tracer adsorbed in mineral soil was stable during the first week before decreasing strongly between 1 and 4 weeks and slightly further between 4 and 8 weeks (Figure S4). Considering that all waters were treated in the same way and that the stability of MMHg (fjord < pond < stream; Figure S3) did not follow the same trend as pH (pond < stream < fjord; Table S1), demethylation most likely explains the observed tracer loss as MMHg is known to be more stable at lower pH. This suggests that the loss of tracer in the solid phase in all slurries with fjord water could be driven by demethylation following the desorption of the tracer in these slurries, continuously changing the equilibrium and leading to the increase in *K*_d_ values over time as compared to the other two waters (Figure 1). It is, however, evident that while the tracer is almost completely demethylated over the course of 8 weeks when dissolved in fjord water (Figure S3), we recovered >35, >50, and >85% of it in the fjord slurries during the adsorption and >50, >55, and >80% during the desorption phase with mineral, stream mineral, and peat particles, respectively (Figure S2; Table S4; Table S5). The higher stability of MMHg in the presence of particles is not surprising as adsorption to particles limits the availability of MMHg for demethylation.^12^ However, since we based our *K*_d_ values on the measurements of MMHg remaining in the solid and the dissolved phases, demethylation occurring during the incubations does not affect our results.

### Partitioning of MMHg between the Solid and Aqueous Phases

The log *K*_d Ads_ values ranged from 1 to 2.5 L kg^–1^ in the slurries with stream mineral and mineral particles and from 1.7 to 3.9 L kg^–1^ in the slurries with peat particles (Table S4), which are comparable to the lower range of previously reported values ranging from 2.2 to 6.06 L kg^–1^.^5,22,28,29^ During adsorption as well as desorption, the highest *K*_d_ values were observed in the fjord water for each solid and in combination with peat in any of the waters (Figure 1, Tables S4 and S5). It is worth mentioning that the peat incubations were performed with 20 ng of tracer per incubation, as compared to 10 ng for the other two solids. The reason for the higher tracer in peat-containing incubations is the higher content of OC and background total Hg (Table S2) to ensure measurable concentrations in the aqueous phase during the desorption phase. If we relate this to the surface area (Table S2), this results in approximately 100, 19, and 15 ng MMHg m^–2^ in the slurries containing peat, stream mineral, and mineral particles, respectively, based on the amount of MMHg added at the beginning of the adsorption phase in all waters. The difference in the ratio of MMHg to surface area did not appear to impact our results as exhaustion of binding sites would lead to higher log *K*_d Ads_ as compared to log *K*_d Des_ values, which is the opposite of what we observed. On the basis of the chemical speciation model, >99% of our tracer was in the form MeHg-DOC in the tracer solutions (Table S6). We can, therefore, rule out that we exhausted the available binding sites in all slurries (Figure 1).

Log *K*_d Des_ values ranged from 1.6 to 2.7 L kg^–1^ in the slurries containing stream mineral and mineral particles and from 2.7 to 4.1 L kg^–1^ in the slurries with peat particles (Table S5). Higher *K*_d_ during desorption compared to adsorption has been observed previously,^22^ and the authors concluded that Hg species appear to be tightly bound and not fully available for desorption unless the equilibrium conditions changed. However, they could not dismiss the possibility that the difference in log *K*_d_ was caused by using different waters (pore water and lake water) during the adsorption and desorption phases. In our experiments, the conditions were kept constant by using the same waters during adsorption and desorption, and thus, we argue that the formation of refractory MMHgP pools explains the higher *K*_d Des_ as discussed further below.

Organic carbon (OC) content, both in the solid^30,31^ and aqueous^32,33^ phases, particle surface area and roughness,^34,35^ pH,^36^ and salinity^34,37^ are known to influence the partitioning of Hg species between the solid and aqueous phases. In our slurries, neither the surface area nor pH appeared to play a major role in controlling partitioning. For the surface area, the measured differences were very small (Table S2) and do not follow the same trend as differences in *K*_d_. In our slurries, pH ranged from 5.0 to 8.2 (Table S3), and no relationship was found between the differences in *K*_d_ and the differences in pH. While increasing pH (up to 5) has been demonstrated to enhance the adsorption of inorganic Hg independently of ionic strength to quartz and gibbsite,^36^ the adsorption of Hg^II^ to microplastics and soil particles was not affected by changes in pH in the range of pH 5 to 9.^34^ While these parameters were not relevant, the interaction of soil OC, DOC, and salinity was found to affect *K*_d_ during adsorption and desorption in a similar way (Figure 2). The greatest impact on *K*_d_ values during both, the adsorption and desorption phases, was associated with the high OC content in the peat (50% as compared to 0.04 and 0.06% in the other two soils; Table S2). This is in line with previous work assessing the effect of soil organic matter removal, which reduced the adsorption capacity of soils by up to 25%.^31^ In addition to *K*_d_ increasing with increasing OC (%) in the solid phase, *K*_d_ was found to decrease with increasing DOC in the aqueous phase in freshwater (Figure 2), emphasizing the role of binding sites associated with OC in MMHg partitioning. However, *K*_d_ was higher in slurries with fjord water for all solids, despite fjord slurries representing intermediate DOC concentrations. The higher *K*_d_ in the slurries with fjord water compared to the slurries with freshwater is likely driven by the demethylation of the aqueous MMHg tracer in fjord water slurries.

**Figure 2:**
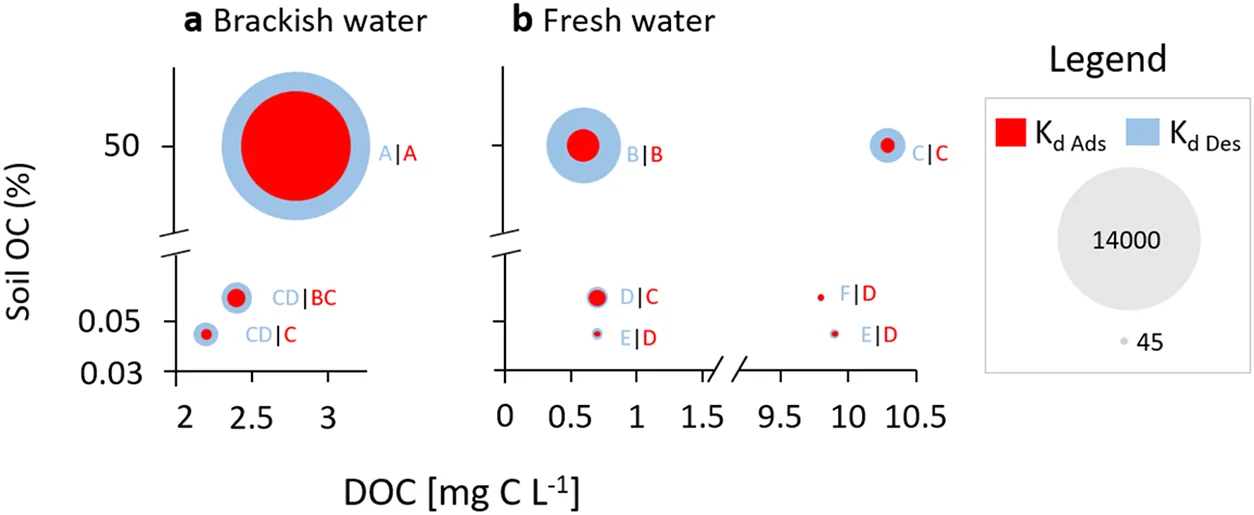
Role of soil OC (log-scaled), DOC, and salinity in *K*_d Des_ and *K*_d Ads_. The bubble sizes represent the *K*_d_ values, and red and blue refer to the adsorption and desorption coefficient at equilibrium (last time point), respectively, as represented by the size of the gray circles and *K*_d_ values in the legend on the right. Systems not connected by the same letters are significantly different based on one-way ANOVA followed by Tukey–Kramer HSD (red during adsorption, blue during desorption).

### Formation of Refractory Pools

By studying the relationship between *K*_d Des_ and *K*_d Ads_, we can observe the formation of refractory MMHg pools in all slurries that we tested (Figure 3). In a fully reversible equilibrium, we would expect *K*_d Des_ to be equal to *K*_d Ads_, with all data to plot on the 1:1 line. As *K*_d Des_ is higher than *K*_d Ads_ (*p* < 0.01), we conclude that not all of the MMHg that adsorbed during the adsorption phase was available for desorption; hence, refractory pools were formed. A higher *K*_d_ during desorption compared with that during adsorption has also been observed previously for different mercury species.^22,31,38^ However, the formation of refractory particle-bound MMHg has not been systematically quantified.

**Figure 3:**
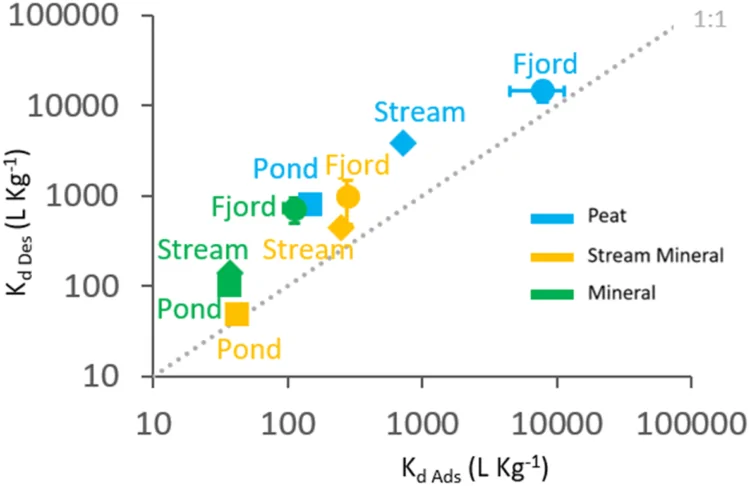
Relationship between *K*_d Des_ and *K*_d Ads_ in the different slurries included in this study. The dashed gray line represents a 1:1 relationship. Data above this line represent the formation of refractory particulate MM^201^Hg.

We found the highest refractory fraction in the slurries containing peat particles, in which 83 (±2.5), 67 (±3.0), and 39 (±30) % of the adsorbed tracer entered the refractory pool in the slurries with stream, pond, and fjord water, respectively. In the slurries with mineral particles, 34 (±4.5), 30 (±1.7), and 35 (±9.8) % of the tracer became refractory in the slurries with stream, pond, and fjord water, respectively. For the slurries with stream mineral particles, we observed 24 (±3.9) and 45 (±30) % in the slurries with stream and fjord water, respectively (no refractory pool was calculated for the stream mineral–pond water slurry as there was no significant difference between *K*_d Des_ and *K*_d Ads_, Figure S5). It should be noted that our estimated refractory pools assume that *K*_d Ads_ reflects the distribution of MMHg between the exchangeable pool on the particle and MMHg in the aqueous phase. Formation of refractory MMHg during the adsorption phase and the short time of incubation used before the water was exchanged to quantify *K*_d Des_ are both factors which would result in our estimates of refractory pools to be underestimated. The presented fraction of refractory MMHg should thus be viewed as lower estimates. Our results support that differences in the formation of refractory pools are linked to OC content in the solids (peat > stream mineral and mineral), whereas water chemistry did not have a significant impact on the formation of refractory MMHg pools (Figure 4). This is in contrast to the clear trend observed for *K*_d_, which in addition to soil OC was influenced by the DOC and salinity (Figure 2). The large uncertainty of refractory MMHgP in the slurries with fjord water (Figure S5) may be related to the demethylation of the tracer in the fjord water leading to higher variability between the treatments compared to the slurries with the other waters.

**Figure 4:**
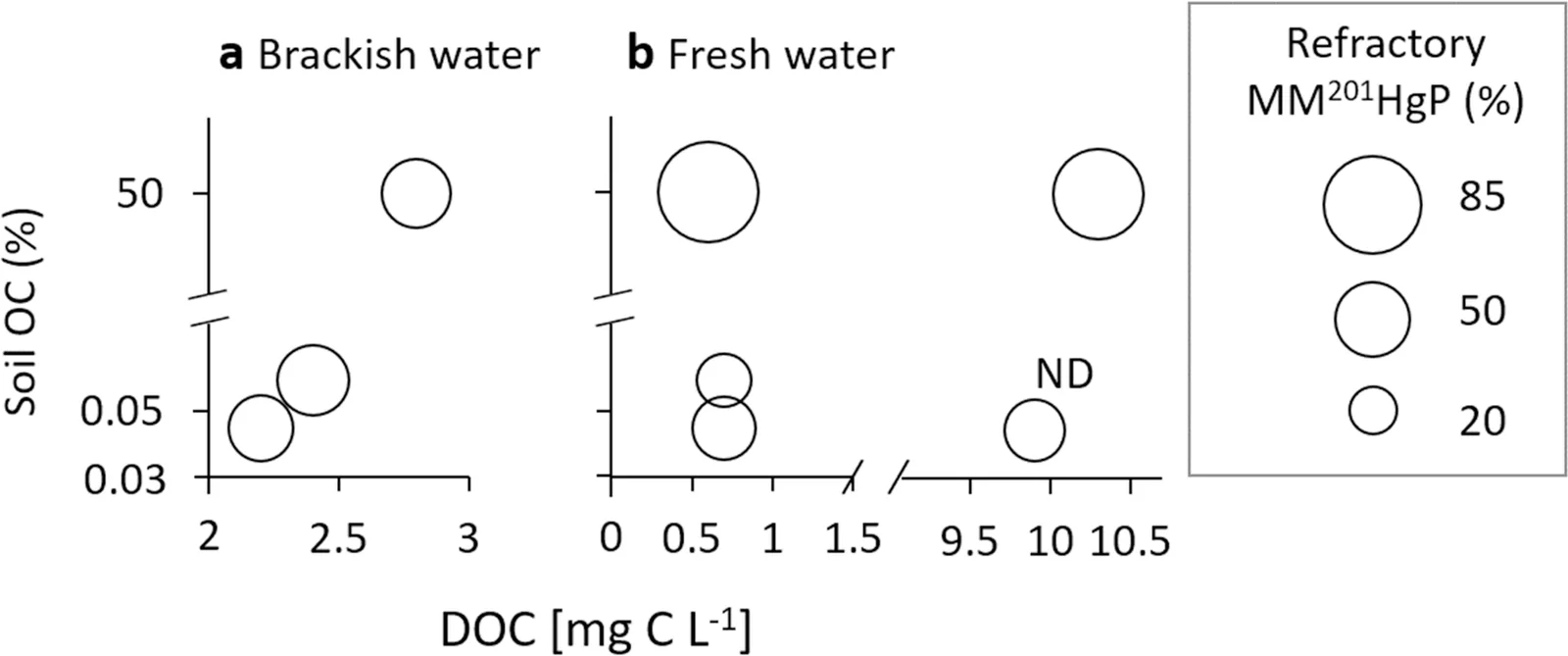
Formation of refractory MMHg pools (%) in relation to DOC in the water and OC in the solids. The bubble sizes represent the (%) values as represented in the legend on the right. Due to no statistical difference between *K*_d Des_ and *K*_d Ads_ in the pond stream mineral slurries, a refractory MMHgP pool was not calculated for this system (ND).

### Mechanistic Discussion

Both chemical and physical processes can alter the availability of ionic Hg species, including MMHg, for desorption and can lead to the formation of refractory pools over time. Although identifying the primary mechanism responsible for this process is beyond the scope of this study, we discuss thermodynamic and physical limitations as potential pathways for the formation of refractory pools. The binding strength of complexing ligands and the relative availability of these could reduce the availability for desorption over time. The stability of MMHg complexed to Cl^–^ or OH^–^ is, for example, much lower than the stability of MMHg when complexed to thiol groups (available on DOM and POM). In our experiment, tracers were pre-equilibrated with natural waters, and chemical speciation modeling indicates that >99.9% was thiol-bound prior to slurry addition. In the slurries, the availability of thiol groups (on POM or DOM) would be even higher, and other potential ligands would remain outcompeted. Given the fact that MMHg ligand exchange, also when bound to thiol groups on natural DOM,^21^ is fast (within a few minutes), we consider it highly unlikely that the formation of refractory pools observed is driven by slow kinetics. Also, since the thermodynamic stability of MMHg bound to different types of reduced sulfur groups on DOM likely is similar (as shown for Hg^II^ as Hg­(NOM-RS)_2_, Hg­(Cys)­(NOM-RS), and Hg­(Cys)_2_),^39^ difference in binding strength is unlikely to be a key driver.

Instead, we argue that the formation of refractory pools is driven by physical limitations, via the encapsulation of MMHg into nano- and micropores in the particles. This process has also been suggested in earlier work, where experimental data indicated the formation of refractory pools of Hg^II^ and MMHg.^22,40^ Furthermore, entrapment of DOM within porous particle structures has been shown to protect it from remineralization, as exoenzymes cannot access these pores.^41,42^ In our experiment, MMHg may enter these structures either bound to small DOM molecules or via migration into particulate binding sites within the pore network. On longer time scales, this occlusion process may be even further enhanced through the buildup of a surface layer, e.g., biofilms.^43^ It is important to note that what we refer to as the refractory pool describes the formation of a refractory pool of a labeled tracer that has recently adsorbed to the particles. The ambient particulate pool of Hg may therefore be even less available for desorption, as a greater fraction of ambient Hg and MMHg may be subject to such physical entrapment over time. The refractory pool of ambient Hg is thus likely to be much larger than that of the tracer. However, the use of ambient waters already containing Hg in the desorption step did not allow us to follow the desorption of ambient Hg.

## Environmental Implications

By directly controlling the availability of Hg to enter the dissolved pool during export, the formation of refractory Hg pools indirectly controls the availability of Hg for biogeochemical transformations as well as biological uptake in downstream systems.^44^ Rivers and coastal oceans are of particular interest, as they serve as pathways through which MMHg is transported from terrestrial to marine environments.^1^ In many fluvial systems, the fraction of MMHgP may constitute the majority of the MMHg in bulk water (e.g., >50% in the Mackenzie River, Canada,^45^ >60% in the Cedar River, USA,^46^ >85% in the Yellow River, China^47^). In addition to geographical differences, the total loadings of MMHgP may vary temporally. For example, the MMHgP export from the Yellow River varied between 1.89 kg year^–1^ during the dry season and 21.3 kg year^–1^ during the humid season in 2018.^48^ These previous observations together with our study of the formation of refractory Hg pools emphasize the large variability of MMHg bioavailability that needs to be accounted for, both geographically and over time, to understand the risks associated with its biological uptake.

Our findings are also important for predicting future trends, as climate change and increasing extreme events are likely to enhance HgP and MMHgP exports to aquatic systems. For example, wildfires can drive rapid inputs of large amounts of particulate matter with a high OC content, ultimately leading to accelerated HgP and MMHgP concentrations downstream of the event.^49^ In this study, we use soils and waters from the Arctic, where high exposure of MMHg is a major concern^6^ and warming is nearly four times faster compared to global averages.^13^ An accelerated export of Hg from northern peatlands is expected as a result of permafrost thaw.^50^ These processes are expected to increase the loadings of HgP to coastal oceans. The data we present here support the assumption^9^ that most of this exported HgP will likely be in a refractory form and stress the importance of further expanding our understanding on the formation of refractory HgP.

## Conclusions

We provide experimental evidence for the formation of refractory particle-bound MMHg in natural soil–water systems resembling a gradient from terrestrially influenced freshwaters to marine systems. Refractory MMHg pools formed in nearly all investigated slurries and accounted for a substantial fraction of the adsorbed MMHg, with the largest refractory fractions observed for the organic-rich peat particles. While soil OC, DOC, and water chemistry strongly influenced MMHg partitioning, their effects on the formation of refractory MMHg pools were less pronounced. In particular, our results indicate that OC is an important factor for refractory pool formation, whereas differences in the water chemistry were negligible.

The formation of refractory particle-bound MMHg limits its return to the dissolved phase and thereby influences its transport, bioavailability, and potential for biological uptake in downstream aquatic systems. Consequently, considering only equilibrium partitioning may not fully capture the environmental fate of particle-bound MMHg exported from the terrestrial environments. Further studies are needed to identify the mechanisms controlling the formation and stability of refractory MMHg pools and quantify refractory fractions of ambient MMHg under environmentally relevant conditions.

## Supplementary Material



## Data Availability

The data supporting this article are available in the Bolin Center Database.^51^

## Notes

More detailed information on the experimental setup including a schematic, environmental matrices used, and quality assurance (PDF)
