# Supplementary material for: Formation of Refractory Monomethylmercury Pools by Particle Adsorption
Source: ACS ES T Water. 2026 Sep 19;6(10):6247–55. doi: 10.1021/acsestwater.6c00243 (PMC13660563; doi:10.1021/acsestwater.6c00243)
Supplement: Supplementary file 1 [file ew6c00243_si_001.pdf]

Supporting Information

Formation of Refractory Monomethylmercury Pools by Particle Adsorption

Sonja Gindorf[1,2], Abhinav Bharadwaj Tirunaghari[1], Charlotte Haugk[1,2], Alyssa Azaroff[1,2], A. Britta K. Sannel[2,3], Sofi Jonsson*[1,2]

[1]*Department of Environmental Science, Stockholm University, 114 18 Stockholm, Sweden*

[2]*Bolin Centre for Climate Research, Stockholm University, 114 18 Stockholm, Sweden*

[3]*Department of Physical Geography, Stockholm University, 114 18 Stockholm, Sweden*

*Corresponding Author: sofi.jonsson@aces.su.se

The supporting Information contains 6 tables and 5 figures.

Supplementary Tables S1

Supplementary Figures S5

Supplementary Tables

Table S1: Water characteristics.

Water	pH	Salinity (unitless)	DOC [mg C L^{-1}]	Latitude (DMS)	Longitude (DMS)
Pond	4.7	N/A	10.0 ± 0.2	68°34'81.3"N	18°96'38.1"E
Stream	7.2	N/A	0.8 ± 0.1	68°36'29.9"N	18°78'65.6"E
Fjord	7.5	8.3	2.3 ± 0.3	70°05'84.8"N	25°01'97.3"E

Table S2: Soil characteristics (THg refers to the total of all Hg forms in the sample).

Soil	OC %	THg (µg g^{-1})	surface area (m^2 g^{-1})	Latitude (DMS)	Longitude (DSM)
Peat	50.17 ± 0.8	20.27	1.3	69°38'44.9"N	24°25'54.3"E
Mineral	0.04 ± 0.02	0.47	1.2	69°89'87.4"N	24°99'72.9"E

Stream Mineral	0.06 ± 0.02	1.72	1.4	68°36'32.6"N	18°78'69.5"E

Table S3: Effect of combining the waters and soils into slurries on water pH and DOC.

Soil Matrix	Water Matrix	pH	DOC [mg C L^{-1}]
Stream Mineral	Fjord	7.3	2.4
Peat	Fjord	5.6	2.8
Mineral	Fjord	7.3	2.0
Stream Mineral	Pond	6.1	9.8
Peat	Pond	5.2	10.3
Mineral	Pond	5.0	9.9
Stream Mineral	Stream	8.2	0.7
Peat	Stream	5.7	0.6
Mineral	Stream	7.1	0.7

Table S4: Adsorption kinetic study (SM= Stream Mineral Sediment; M= Mineral; P=Peat). n_{aq} = number of aqueous samples, n_{solid} = number of solid samples.

Soil	Water	Time point	Time (hours)	n_{aq}	n_{solid}	$MM^{201}Hg_{solid}$ [ng g^{-1}]	$MM^{201}Hg_{aq}$ [ng L^{-1}]	Recovery $MM^{201}Hg$ (%)	Log $K_{d\ Ads}$ [L Kg^{-1}]
SM	Fjord	T0	0.25	1	0		141	70	
SM	Fjord	T1	0.35	1	1	2.28	73.8	62	1.49
SM	Fjord	T2	0.6	1	0		53.0		
SM	Fjord	T3	1	1	0		45.6		
SM	Fjord	T4	3	1	1	2.77	46.5	51	1.77
SM	Fjord	T5	24	1	1	3.56	14.8	47	2.38
SM	Fjord	T6	48	3	1	4.21	11.4±1.40	54	2.50
SM	Pond	T0	0.35	1	0		206	99	
SM	Pond	T1	0.5	1	1	1.96	170	103	1.06
SM	Pond	T2	0.7	1	0		156	75	
SM	Pond	T3	1	1	0		145	69	
SM	Pond	T4	3	1	1	2.96	174	112	1.23
SM	Pond	T5	24	1	1	3.27	151	107	1.33
SM	Pond	T6	48	3	2	3.98±0.31	109±28.3	96±20	1.61±0.04
SM	Stream	T0	0.25	1	0		198	98	
SM	Stream	T2	0.7	1	0		86.1		
SM	Stream	T3	1	1	0		74.0		
SM	Stream	T4	3	1	1	4.65	53.7	77	1.94
SM	Stream	T5	24	1	1	6.57	27.3	80	2.38
SM	Stream	T6	47	3	2	6.33±0.09	25.4±0.69	79±1	2.40±0.01
M	Fjord	T0	0.25	1	0		135	69	
M	Fjord	T1	0.4	1	1	1.47	149	83	0.99
M	Fjord	T2	0.7	1	0		121		
M	Fjord	T3	1	1	0		101		
M	Fjord	T4	3	1	1	2.58	95.5	61	1.43
M	Fjord	T5	24	1	1	3.97	55.5	47	1.85
M	Fjord	T6	47	3	2	3.76±0.41	33.6±1.47	36±1	2.04±0.07
M	Pond	T0	0.3	1	0		212	101	
M	Pond	T1	0.5	1	1	2.03	199	104	1.01
M	Pond	T2	0.8	1	0		171		
M	Pond	T3	1	1	0		169		
M	Pond	T4	3	1	1	3.66	194	112	1.28
M	Pond	T5	25	1	1	5.11	179	112	1.46
M	Pond	T6	48	3	2	5.30±0.06	145±1.87	98±1	1.57±0.00

M	Stream	T0	0.3	1	0		151	74	
M	Stream	T1	0.45	1	1	1.36	183	97	0.87
M	Stream	T2	0.7	1	0		120		
M	Stream	T3	1	1	0		177		
M	Stream	T4	3	1	1	1.73	147	81	1.07
M	Stream	T5	24	1	1	2.51	137	81	1.26
M	Stream	T6	47	3	2	2.31±0.15	66.4±9.16	40±4	1.58±0.02
P	Fjord	T0	0.4	1	0	0.00	275	67	
P	Fjord	T1	0.3	1	1	29.55	48.1	85	2.79
P	Fjord	T2	0.8	1	0		17.1		
P	Fjord	T3	1	1	0		12.4		
P	Fjord	T4	3	1	1	26.74	17.7	73	3.18
P	Fjord	T5	24	1	1	34.25	4.96	85	3.84
P	Fjord	T6	48	3	2	34.44±1.74	4.44±2.02	87±3	3.85±0.20
P	Pond	T0	0.5	1	0	0.00	406.4	97	
P	Pond	T1	0.4	1	1	13.72	283	102	1.69
P	Pond	T2	0.7	1	0		292		
P	Pond	T3	1	1	0		253		
P	Pond	T4	3	1	1	15.53	311	113	1.70
P	Pond	T5	24	1	1	22.09	206	105	2.03
P	Pond	T6	48	3	2	24.93±0.34	169±10.2	103±3	2.16±0.02
P	Stream	T0	0.5	1	0	0.00	307	76	
P	Stream	T1	0.4	1	1	20.10	155	90	2.11
P	Stream	T2	0.7	1	0		101		
P	Stream	T3	1	1	0		107		
P	Stream	T4	3	1	1	20.85	77.6	72	2.43
P	Stream	T5	24	1	1	28.65	49.0	82	2.77
P	Stream	T6	48	3	2	29.79±3.07	42.0±3.01	84±8	2.86±0.01

Table S5: Desorption kinetic study (SM= Stream Mineral Sediment; M= Mineral; P=Peat). n_{aq} = number of aqueous samples, n_{solid} = number of solid samples.

Soil	Water	Time point	Time (hours)	n_{aq}	n_{solid}	$MM^{201}Hg_{solid}$ [ng g^{-1}]	$MM^{201}Hg_{aq}$ [ng L^{-1}]	Recovery $MM^{201}Hg$ (%)	Log $K_{d\ Des}$ [L Kg^{-1}]
SM	Fjord	T0	0	0	1	4.21	0.00		
SM	Fjord	T1	4	1	0		10.6		
SM	Fjord	T2	24	1	1	3.37	8.48	89	2.61
SM	Fjord	T3	193	1	1	2.90	6.18	76	2.69
SM	Fjord	T4	336	1	0		4.94		
SM	Fjord	T5	669	1	1	3.16	3.76	79	2.95
SM	Fjord	T6	1537	3	3	2.28±0.04	3.08±1.10	58±2	2.94±0.20
SM	Pond	T0	0	0	1	3.98	0.00		
SM	Pond	T1	4	1	0		29.9		
SM	Pond	T2	24	1	1	1.85	27.8	79	1.86
SM	Pond	T3	193	1	1	1.49	39.4	86	1.60
SM	Pond	T4	336	1	0		36.4		
SM	Pond	T5	669	1	1	1.53	33.1	75	1.69
SM	Pond	T6	1537	3	3	1.44±0.16	31.1±1.16	71±5	1.69±0.04
SM	Stream	T0	0	0	1	6.33	0.00		
SM	Stream	T1	4	1	0		16.1		
SM	Stream	T2	24	1	1	4.70	17.4	88	2.44
SM	Stream	T3	192	1	1	4.40	16.9	82	2.43
SM	Stream	T4	336	1	0		13.7		
SM	Stream	T5	669	1	1	3.98	12.4	72	2.52
SM	Stream	T6	1537	3	3	3.76±0.04	9.04±0.48	66±1	2.64±0.02
M	Fjord	T0	0	0	1	3.76	0.00		
M	Fjord	T1	3	1	0		16.95		
M	Fjord	T2	24	1	1	2.17	18.25	107	2.08
M	Fjord	T3	192	1	1	2.57	7.64	90	2.53
M	Fjord	T4	336	1	0		4.06		
M	Fjord	T5	670	1	1	2.53	5.27	83	2.68

M	Fjord	T6	1534	3	3	1.57±0.13	2.40 ±0.78	50±4	2.8 4±0.13
M	Pond	T0	0	0	1	5.30	0.00		
M	Pond	T1	3	1	0		8.18		
M	Pond	T2	24	1	1	2.97	21.041	96	2.21
M	Pond	T3	334	1	1	2.58	30.5	104	1.97
M	Pond	T4	482	1	0		26.9		
M	Pond	T5	817	1	1	2.42	29.5	98	1.95
M	Pond	T6	1677	3	3	2.64±0.17	28.0±2.7	100±8	2.02±0.02
M	Stream	T0	0	0	1	2.31	0.00		
M	Stream	T1	3	1	0		9.14		
M	Stream	T2	24	1	1	1.26	4.88	76	2.53
M	Stream	T3	192	1	1	1.00	7.98	77	2.17
M	Stream	T4	336	1	0		14.6		
M	Stream	T5	670	1	1	1.06	12.6	98	1.97
M	Stream	T6	1535	3	3	1.15±0.15	9.43±0.64	89±9	2.14±0.04
P	Fjord	T0	0	0	1	34.44	0.00		
P	Fjord	T1	3	1	0		2.21		
P	Fjord	T2	24	1	1	33.0	6.89	98	3.68
P	Fjord	T3	192	1	1	30.0	3.52	88	3.93
P	Fjord	T4	336	1	0		2.72		
P	Fjord	T5	677	1	1	30.3	1.97	88	4.19
P	Fjord	T6	1536	3	3	27.7±0.29	2.07±0.53	81±1	4.15±0.12
P	Pond	T0	0	0	1	24.9	0.00		
P	Pond	T1	3	1	0		13.6		
P	Pond	T2	24	1	1	21.9	37.4	102	2.77
P	Pond	T3	192	1	1	19.9	45.8	97	2.64
P	Pond	T4	336	1	0		47.1		
P	Pond	T5	677	1	1	22.1	38.1	103	2.77
P	Pond	T6	1536	3	3	20.3±0.42	24.9±0.42	91±1	2.91±0.02
P	Stream	T0	0	0	1	29.79	0.00		
P	Stream	T1	3	1	0		4.60		
P	Stream	T2	24	1	1	29.88	8.27	103	3.56
P	Stream	T3	192	1	1	30.00	9.96	104	3.48
P	Stream	T4	336	1	0		6.01		
P	Stream	T5	677	1	1	30.39	10.4	105	3.47
P	Stream	T6	1536	3	3	30.60±0.91	8.18±0.10	105±3	3.57±0.01

Table S6: Chemical Speciation Model of $MM^{201}Hg$ in the tracer solutions (50µg L^{-1}). Salinity was not measured in the Pond and Stream waters and assumed to be 1 and 2, respectively. We used the thermodynamic formation constants (Log K) of 5.4 for MMHgCl[1], and 16.1 to 17.4 for MMHg-DOC (aq) with the concentrations of MMHg, Cl^- (based on salinity), and thiols (calculated as 0.15 % * DOC)[2,3].

Water	[Cl^-] (M)	[DOC] (mg L^{-1})	[DOC-SH] (nM)	MeHg-Cl (%)	MeHg-DOC (%)
Pond	0.02	10	469	< 0.01	> 99.9
Stream	0.03	0.8	37.5	<0.01	>99.9
Fjord	0.14	2.3	108	<0.01	>99.9

Supplementary Figures

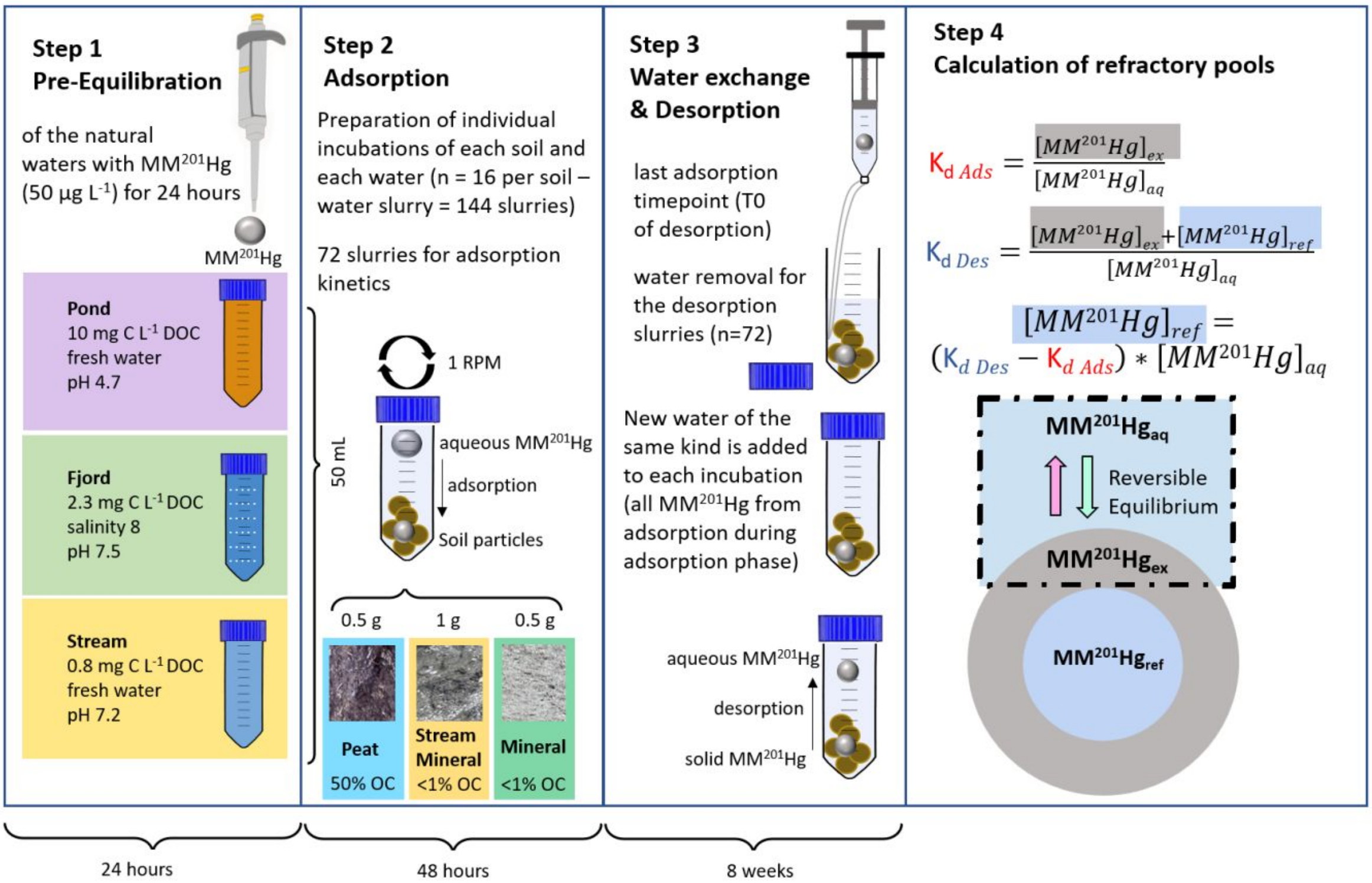

Figure S1: Schematic of the experimental setup and workflow.

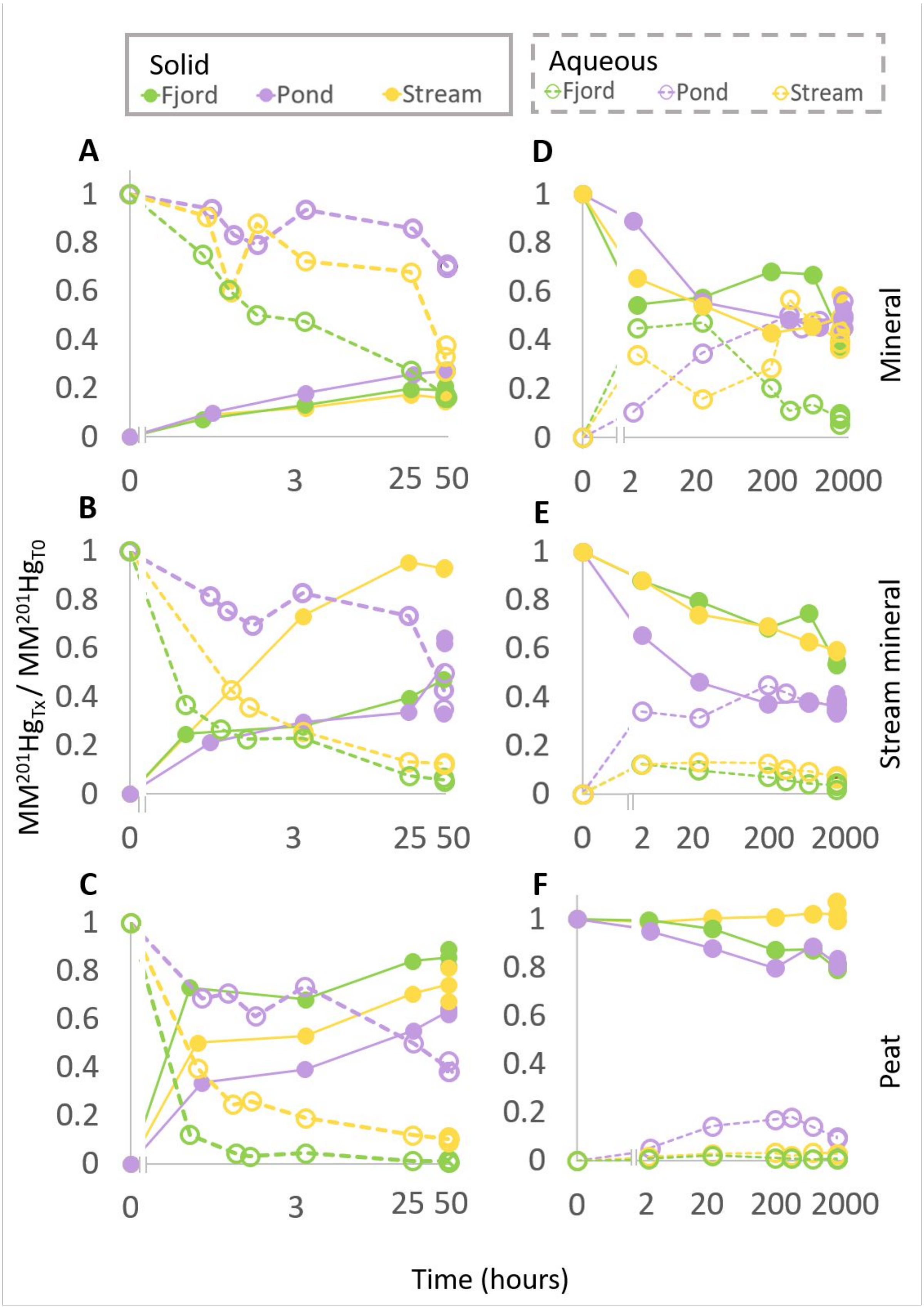

Figure S2: Adsorption (A,B,C) and desorption (D, E, F) kinetics for the different particles over time (log-transformed). For adsorption studies, the reference T_0 for the relative amount of $MM^{201}Hg$ in the

different matrices is the total added amount (10 ng for mineral (A) and stream mineral (B) slurries, respectively and 20 ng for organic (C)). At the last time point of the adsorption phase, we analyzed duplicates in the solid and triplicates in the aqueous phase (from 3 separately prepared incubations). For the desorption studies, the reference T_0 for the relative amount of $MM^{201}Hg$ in the different matrices is the total amount of $MM^{201}Hg$ in the soil at the last time point of the adsorption phase. At the last time point of the desorption phase, we analyzed triplicates in the solid and aqueous phase (from 3 separately prepared incubations).

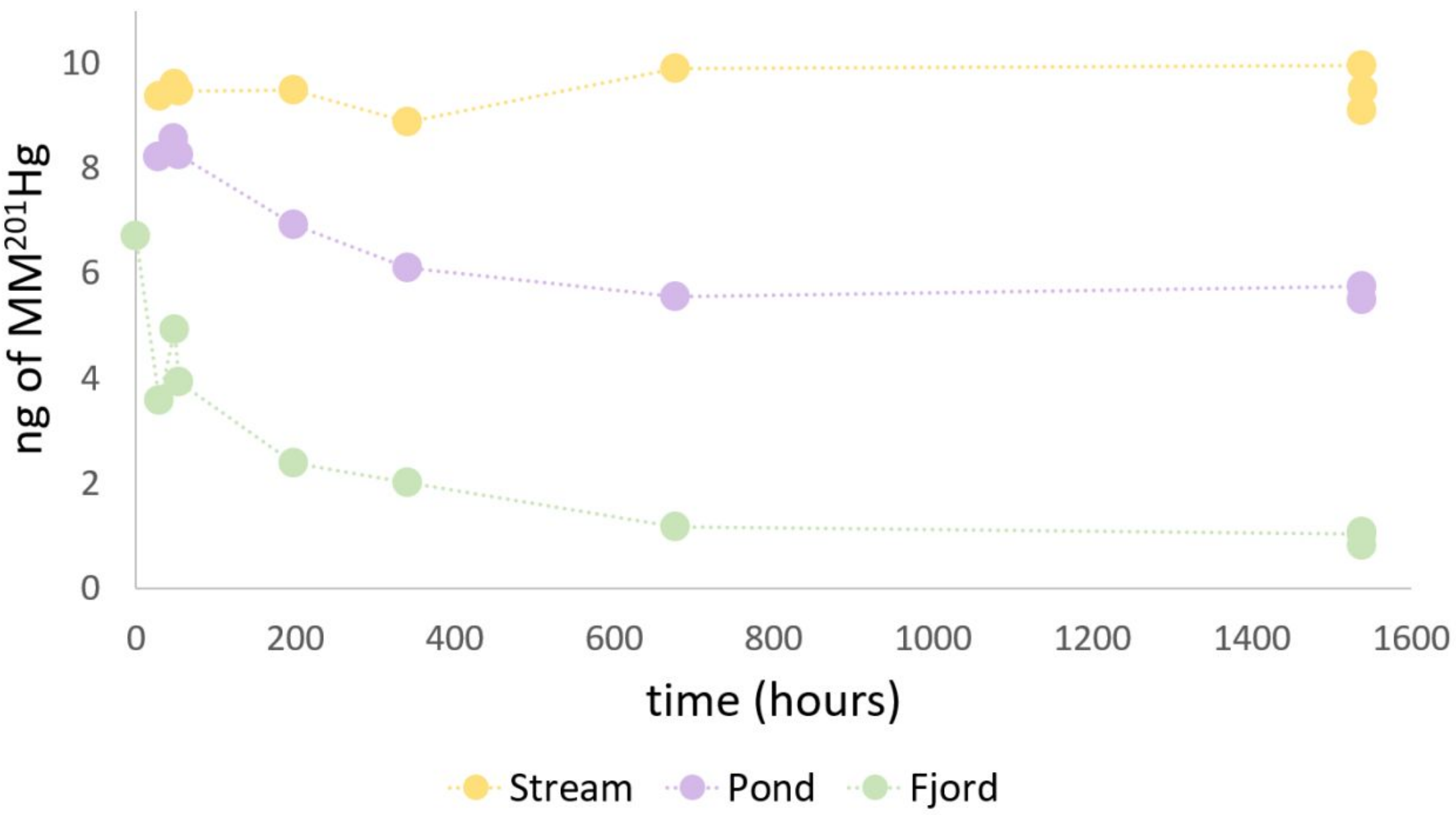

Figure S3: Stability of the tracer in each of the waters over 8 weeks.

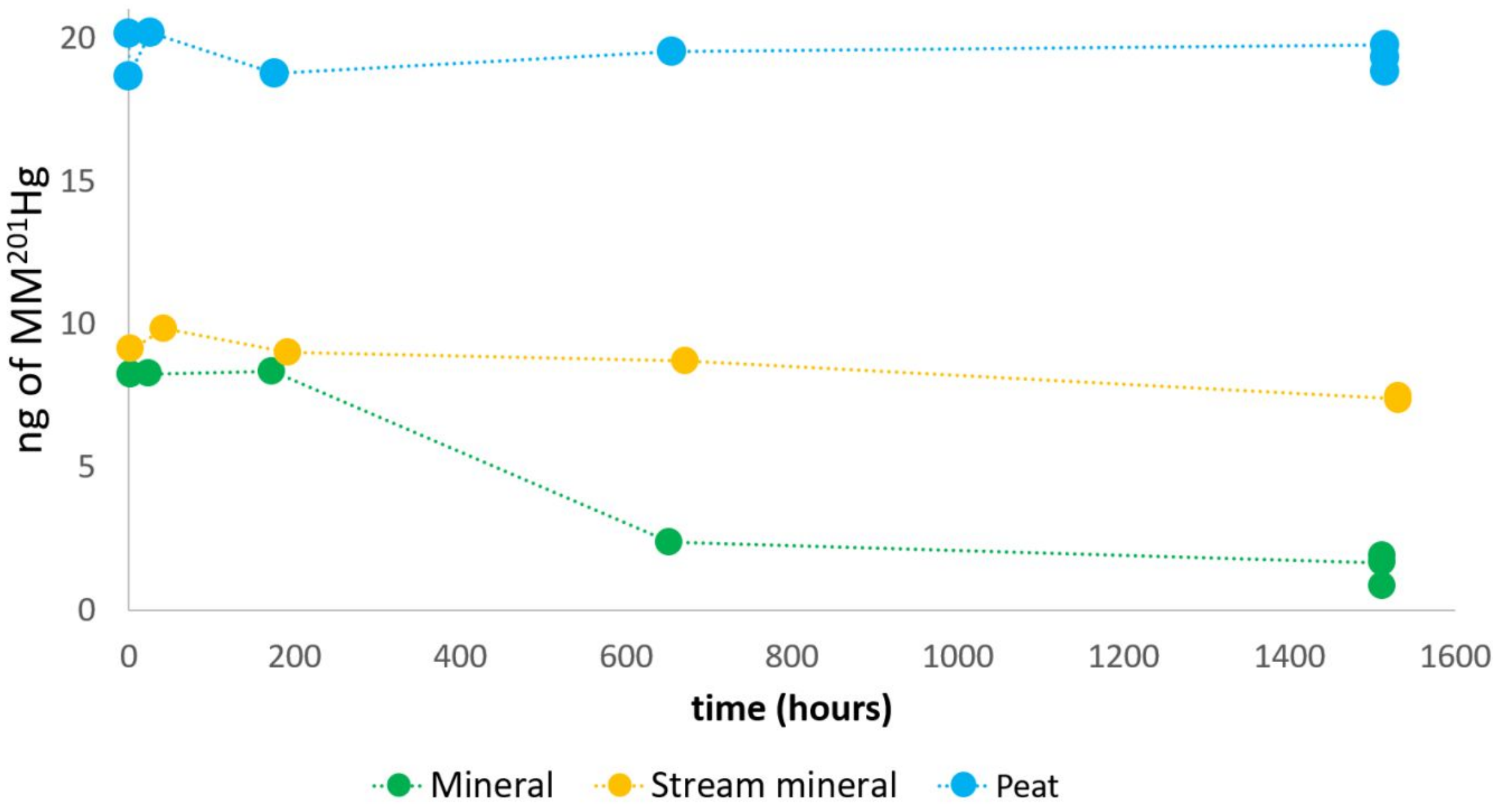

Figure S4: Stability of the tracer in each of the soils over 8 weeks (note that for peat 20 ng of tracer were added, whereas for mineral and stream mineral particles, 10 ng were added).

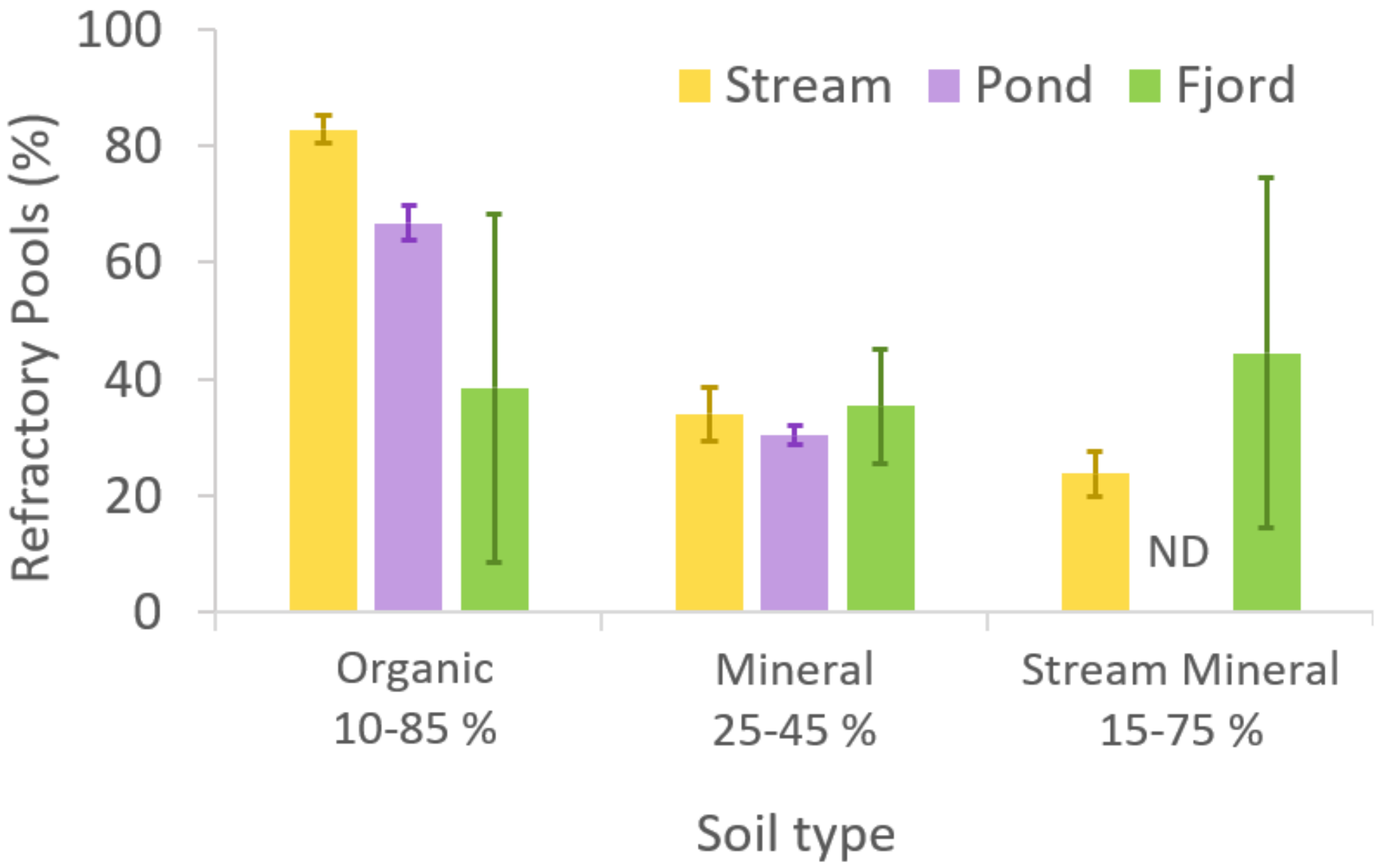

Figure S5: The relative formation of refractory MMHg tracer in the different soil-water slurries. Error bars represent the standard deviation of calculating the refractory pool of all combinations of $K_{d\ Des}$ (n=3) and $K_{d\ Ads}$ (n=2) for each system. A refractory pool was not calculated for Stream Mineral Pond slurries due to the lack of statistical difference between $K_{d\ Des}$ and $K_{d\ Ads}$ in these slurries (ND).

References

1 E. Seelen, V. Liem-Nguyen, U. Wünsch, Z. Baumann, R. Mason, U. Skyllberg and E. Björn, Dissolved organic matter thiol concentrations determine methylmercury bioavailability across the terrestrial-marine aquatic continuum, *Nat. Commun.*, 2023, **14**, 6728.

2 V. Liem-Nguyen, U. Skyllberg and E. Björn, Thermodynamic Modeling of the Solubility and Chemical Speciation of Mercury and Methylmercury Driven by Organic Thiols and Micromolar Sulfide Concentrations in Boreal Wetland Soils, *Environ. Sci. Technol.*, 2017, **51**, 3678–3686.

3 U. Skyllberg, Competition among thiols and inorganic sulfides and polysulfides for Hg and MeHg in wetland soils and sediments under suboxic conditions: Illumination of controversies and implications for MeHg net production, *J. Geophys. Res. Biogeosciences*, 2008, **113**, 1–14.
